# Distribution and Genetic Diversity of Grapevine Viruses in Russia

**DOI:** 10.3390/plants10061080

**Published:** 2021-05-27

**Authors:** Elena Porotikova, Uliana Terehova, Vitalii Volodin, Eugeniya Yurchenko, Svetlana Vinogradova

**Affiliations:** 1 Institute of Bioengineering, Research Center of Biotechnology of the Russian Academy of Sciences, Leninsky Prospect 33, 119071 Moscow, Russia; plantvirus@mail.ru (E.P.); dmitrenko_uliana@mail.ru (U.T.); 2 All-Russian National Scientific Research Institute of Vine and Wine Growing “Magarach” Ras, Str. Kirova 31, 298600 Yalta, Crimea; vitaliivolodin1988@gmail.com; 3 North Caucasian Regional Research Institute of Horticulture and Viticulture, 40 Years of Victory Street 39, 350072 Krasnodar, Russia; yug.agroekos@yandex.ru

**Keywords:** *Vitis vinifera*, grapevine viruses, grapevine virus infection, viral diagnostics, phylogenetic analysis, molecular variants, genetic variability, RFLP

## Abstract

Viral diseases can seriously damage the vineyard productivity and the quality of grape and wine products. Therefore, the study of the species composition and range of grapevine viruses is important for the development and implementation of strategies and tactics to limit their spread and increase the economic benefits of viticulture. In 2014–2019, we carried out a large-scale phytosanitary monitoring of Russian commercial vineyards in the Krasnodar region, Stavropol region and Republic of Crimea. A total of 1857 samples were collected and tested for the presence of *Grapevine rupestris stem pitting-associated virus* (GRSPaV), *Grapevine virus A* (GVA), *Grapevine leafroll-associated virus-1* (GLRaV-1), *Grapevine leafroll-associated virus-2* (GLRaV-2), *Grapevine leafroll-associated virus-3* (GLRaV-3), *Grapevine fanleaf virus* (GFLV), and *Grapevine fleck virus* (GFkV) using RT-PCR. Out of all samples tested, 54.5% were positive for at least one of the viruses (GRSPaV, GVA, GLRaV-1, GLRaV-2, GLRaV-3, GFLV, GFkV) in the Stavropol region, 49.8% in the Krasnodar region and 49.5% in the Republic of Crimea. Some plants were found to be infected with several viruses simultaneously. In the Republic of Crimea, for instance, a number of plants were infected with five viruses. In the Krasnodar region and the Republic of Crimea, 4.7% and 3.3% of the samples were predominantly infected with both GFkV and GRSPaV, whereas in the Stavropol region, 6% of the selected samples had both GLRaV-1 and GVA infections. We carried out a phylogenetic analysis of the coat protein genes of the detected viruses and identified the presence of GVA of groups I and IV, GRSPaV of groups BS and SG1, GLRaV-1 of group III, GLRaV-2 of groups PN and H4, GLRaV-3 of groups I and III. The results obtained make it possible to assess the viral load and the distribution of the main grapevine viruses on plantations in the viticultural zones of Russia, emphasizing the urgent need to develop and implement long-term strategies for the control of viral diseases of grapes.

## 1. Introduction

A large variety of fruits and berries provide humans with beneficial substances necessary for the vital activities of the body; moreover, some cultures, including grapes which is one of the most ancient and valuable fruit crops [1], are the driving force in the economy of a number of southern countries. In 2018, the area occupied by grape plantations throughout the world comprised about 7.4 million hectares (according to OIV-2019, [2]). In Russia, there were 95.9 thousand hectares of grapevine plantations in 2019 (according to the Russian Statistics Service, [3]). However, viticulture all over the world is seriously affected by viral diseases which, depending on the type of virus and the degree of its impact on productivity and product quality, result in losses of $25,000 to $40,000 per hectare [4].

Among woody plants worldwide, grapevine is the culture which is most affected by viral phytopathogens [5]. To date, more than 80 viral and virus-like agents that belong to different taxonomic units are known [6]. The main ones are representatives of the families *Closteroviridae, Betaflexiviridae, Caulimoviridae, Geminiviridae*, and *Secoviridae*. About half of viral phytopathogens are associated with four main disease complexes known as infectious degeneration and decline (fanleaf), leafroll, rugose wood, and fleck [1,7]. The most prevalent grape diseases in the world are grapevine fanleaf caused by *Grapevine fanleaf virus* (GFLV) and grapevine leafroll disease, the most significant infectious agents of which are *Grapevine leafroll-associated virus-1* (GLRaV-1), *Grapevine leafroll-associated virus-2* (GLRaV-2) and *Grapevine leafroll-associated virus-3* (GLRaV-3) [8,9,10]. The main role in the spread of these viruses is played by the vegetative propagation of crops, grafting and insect carriers [11]. It is known that infectious degeneration and leafroll diseases reduce the rate of photosynthesis processes, water exchange, affect the size of berries and contribute to a decrease in grape yield [11,12,13].

Viruses that cause rugose wood include *Grapevine rupestris stem pitting-associated virus* (GRSPaV), *Grapevine virus A* (GVA), *Grapevine virus B* (GVB), *Grapevine virus D* (GVD), *Grapevine virus E* (GVE), and *Grapevine virus F* (GVF). They are characterized by the presence of rupestris stem pitting, kober stem grooving, corky bark, and grooving and pitting of the woody cylinder [7]. Infected grapevines weaken and die within a few years after infection [7].

*Grapevine fleck virus* (GFkV) is one of four representatives of the fleck complex, along with *Grapevine asteroid mosaic-associated virus* (GAMaV), *Grapevine rupestris vein feathering virus* (GRVFV), and *Grapevine red globe virus* (GRGV) [7]. The pathogenicity of GFkV and its direct effect on the viability and productivity of plants have not been sufficiently studied, however, it is known to lead to deterioration of the main organelles of the cell and, along with other viruses, the chemical composition of berries [14,15].

Since fighting against viral phytopathogens is difficult and the ways to completely destroy viruses directly in the vineyard are unknown, it is necessary to use healthy planting material tested for viruses at the stage of seedling production in nurseries [5].

Early detection of foci of viral infection of grapevines allows to prevent their large-scale spreading and take measures to reduce the economic damage to the industry [6]. In Russia, however, insufficient attention was paid to the study of the viral distribution and reduction in grape yield caused by viral infections. In our earlier studies, we have identified GVA, GRSPaV, GLRaV-1, GLRaV-2, and GLRaV-3 in the commercial vineyards in Russia [16,17,18,19,20], although their genetic diversity which is necessary for understanding the evolutionary processes of viruses and finding effective countermeasures still remained unexplored.

The aim of this work was to study the distribution and genetic diversity of grapevine viruses in Russia. Among them, we selected 7 pathogens that have the most significant economic effect and carried out a large-scale phytosanitary study of grapevine plantations in three southern regions of Russia. Five viruses we previously detected were included to understand how widespread they are, as well as GFLV and GFkV, which are usually included in the monitoring of vineyards for viruses.

## 2. Results

### 2.1. Phytosanitary Monitoring

During the studies, we carried out phytosanitary monitoring of 335 grape plantations in various agricultural landscapes, including commercial, small farms, and abandoned plantations. In total, we examined grape plantations of 83 cultivars and hybrids, as well as a number of unspecified cultivars.

**Figure 1:**
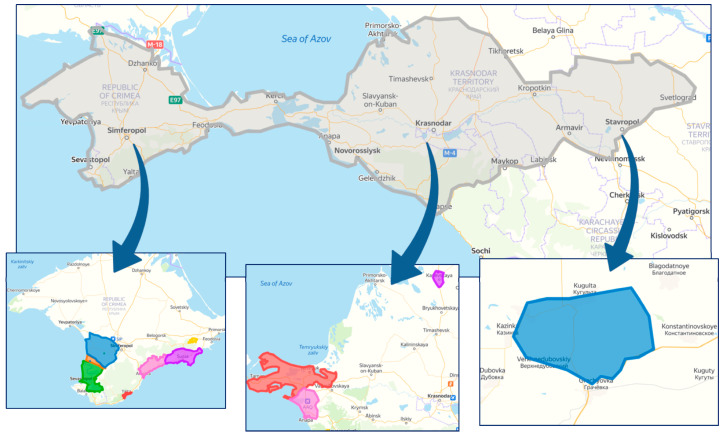
Viticultural zones in the south of Russia, where phytosanitary monitoring was carried out.

Figure 1 shows maps of the surveyed areas. During monitoring, we collected and analyzed using RT-PCR 1857 samples; 1232 were collected in the Crimea, 592 in the Krasnodar region and 33 samples in the vineyards of the Stavropol region which is explained by smaller areas of sheltering vineyards in the Stavropol region.

An analysis of the infection symptoms in the plants of the surveyed territories revealed that over 56% of the samples collected in the Crimea had various types of chlorotic leaf yellowing and spots, which may be a manifestation of infection with various viruses and viroids. The second most common symptom (about 17%) was reddening of the interveinal areas of the leaf blade with main veins remaining green [5]. The described symptom may indicate the presence of grapevine leafroll disease in the vineyard. In the Krasnodar region, the manifestation of symptoms in the form of leaf reddening comprised about 15%, while chlorosis was observed in only 20% of the collected samples. In the Stavropol region, the symptoms of viral diseases were not observed in most of the surveyed plantations. However, a small number of plants had a slight deformation of the leaf blade on the lower layer, which is likely to be associated with the use of pesticides, as well as rolling of some leaves with most of the leaves of the plant remaining green. In addition to the listed symptoms, in the vineyards of the Krasnodar region and Crimea we noted a large number of plants with symptoms of severe leaf roll, deformation of leaves, and zigzag shoots. Such symptoms are characteristic of grapevine infection with GFLV and other nepoviruses, as well as phytoplasma [21]. Often we observed complete wilting and drying out of plants and on the trunks of some grapevines-pits and grooves, which indicates infection of plants with diseases of the rugose wood complex.

**Figure 2:**
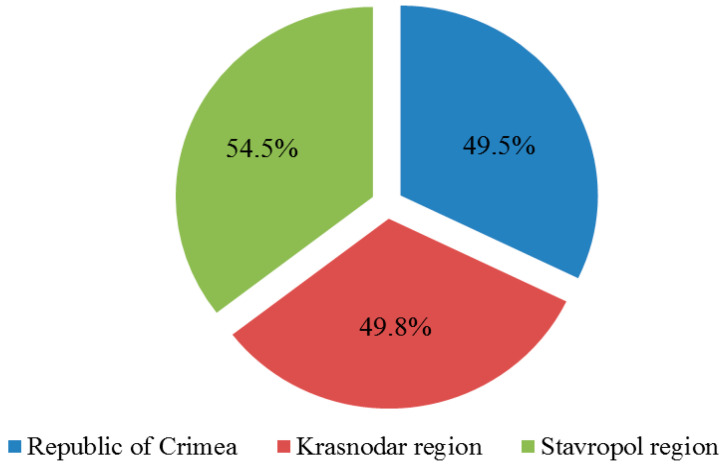
The number of affected grape plants in the vineyards of the surveyed territories (percentage of the total number of samples collected in the region).

Diagnostics of collected samples for the presence of 7 grapevine viruses showed that in the Crimea 610 plants (49.5%) were infected with at least one of the viruses under study, in the Krasnodar region—295 plants (49.8%) and in the Stavropol region—18 plants (54.5%) (Figure 2).

**Figure 3:**
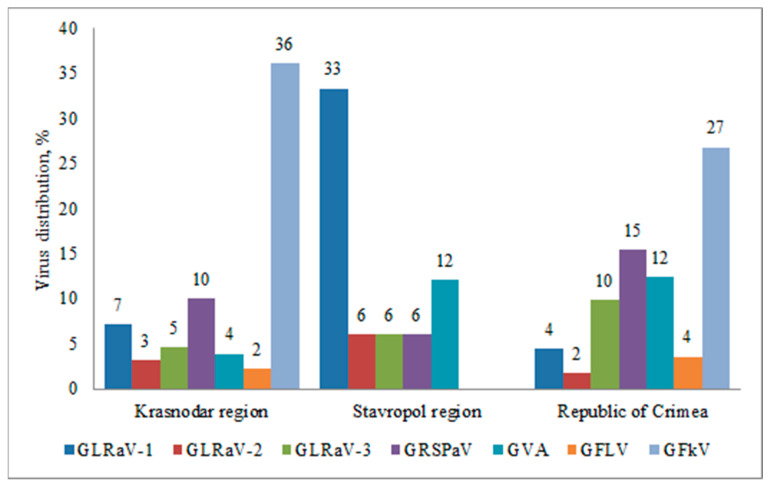
Distribution of grapevine viruses in various regions of the Russian Federation (percentage of the number of samples collected in the region).

In the Krasnodar region and Crimea, we detected all viruses under study, in the Stavropol region—5 out of 7 (GFLV and GFkV were not detected) (Figure 3). This result can be explained by significantly lower number of samples collected in Stavropol region. GFkV was the most prevalent virus in the Krasnodar region and the Republic of Crimea; it was detected in 36% and 27% of the plants, respectively. GFLV was found in the Republic of Crimea and Krasnodar region in 4% and 2% of samples, respectively.

In the Stavropol region, GLRaV-1 was more widespread; it was identified in 33% of the collected samples, while GLRaV-2 and GLRaV-3 were detected in 6% of the plants. This was the highest percentage of GLRaV-2 infections in all surveyed areas. The highest percentage of GLRaV-3 was observed in the Republic of Crimea, where it was found in 10% of the samples. In general, leafroll viruses were less prevalent than viruses belonging to the stem pitting complex.

GRSPaV associated with grapevine rupestris stem pitting disease was identified in 15% of the samples in the Crimea, in 10% of the plants in the Krasnodar region, and in 6% of the samples in the Stavropol region. GVA was more prevalent in the Stavropol region and the Republic of Crimea, where it was detected in 12% of the plants, while in the Krasnodar region the virus was detected only in 4% of the tested samples.

Some of the plants were infected with two or more viruses at the same time (Supplementary Figures S1–S3). In the Crimea and Krasnodar region, the most prevalent mixed infection was infection with both GFkV and GRSPaV; there were about 3.3 and 4.7% of such plants, respectively. In the Stavropol region, 6% of the plants were infected with GVA and GLRaV-1. A small percentage of the samples (0.08–0.16% of the total number of infected plants depending on the virus combination) in the Republic of Crimea were simultaneously infected with five viruses under study. In the Krasnodar region 0.17% of samples were simultaneously infected with GLRaV-1, GRSPaV, GVA, GFkV or GLRaV-1, GLRaV-2, GRSPaV, GFkV.

### 2.2. Phylogenetic Studies and Determination of Molecular Groups of Viruses

Since phylogenetic reconstruction can be affected by recombination [22], a recombination analysis of genes encoding coat proteins was carried out for each of the viruses under study. An analysis in the RDP v.4.100 program did not identify any recombination points in the coat protein genes of the Russian isolates GRSPaV, GVA, GLRaV-1, GLRaV-2, GLRaV-3, GFLV, and GFkV.

### 2.3. Viruses of Rugose Wood Complex

According to phylogenetic analysis of the coat protein gene, GVA isolates are usually classified into 4 groups [23]. Alignment of the GVA coat protein gene sequences using the BLASTn algorithm showed that the identity of the Russian isolates with the GenBank database sequences was 87.52–98.8%. Phylogenetic relationships of GVA isolates were analyzed using the maximum likelihood estimation and presented in the form of a dendrogram (Supplementary Figure S4). An analysis of the nucleotide sequences of the GVA coat protein genes showed that the isolates we found belong to molecular groups I and IV. Group I included most of the isolates from the Crimea which clustered alongside genetic variants from China, Spain, Greece, Jordan, Russia, and USA. Group IV included the isolates from the Crimea and Krasnodar region which clustered alongside isolates from the USA and Greece.

In recent decades, a large number of studies on the genetic diversity of GRSPaV have been carried out, which made it possible to identify several molecular groups of GRSPaV [23,24,25,26]. For instance, according to studies by Sabella et al. (2018), there are four molecular groups that were named according to the corresponding representative isolates [23]. It is noteworthy that mixed infection of several groups is often observed [26]. Our analysis showed that the Russian isolates belong to two groups (Supplementary Figure S5). Most of the isolated samples belong to the GRSPaV-BS group; the viruses currently detected in the Krasnodar region and Stavropol region belong to a single phylogenetic group, in contrast to the Crimean isolates which belong to 2 genetic groups: GRSPaV-BS and GRSPaV-SG1. Using the BLASTn analysis of the coat protein genes of the Russian isolates along with GenBank isolates, we found their identity with the GRSPaV nucleotide sequences at the level of 93.39–98.8%.

### 2.4. Fleck Complex and Grapevine Fanleaf Viruses

A BLASTn analysis for a fragment of the GFkV coat protein gene showed that the identity of the nucleotide sequences of the five Russian isolates with isolates from the GenBank database varied from 88.38% to 100% (Supplementary Figure S6); the closest were isolates from Poland, which was supported by a bootstrap value of 96%. The isolates we found belonged to different clades and were evenly distributed over the phylogenetic tree.

A comparison of fragments of the GFLV coat protein gene using BLASTn demonstrated that the sequences of the Russian isolates are 86.77–90.12% identical with the sequences of the GFLV coat protein gene available in the GenBank. Supplementary Figure S7 shows fragments of phylogenetic trees constructed using the Crimean isolates and all sequences of the GFLV coat protein gene available in the GenBank. As can be seen from the dendrogram, the Russian isolates are divided into three clusters. The first cluster is formed by the Russian isolate 308 together with closely located samples from the USA and Spain. The second cluster includes the Crimean isolate 311 and several isolates from the Czech Republic and Italy. The third cluster is comprised by two other Russian isolates with a high bootstrap support (99%) clustered next to samples from Turkey and Tunisia.

### 2.5. Grapevine Leafroll Disease Viruses

According to previous studies in the field of GLRaV-1 phylogeny, all isolates belong to 3 groups [23]. We analyzed the nucleotide sequences of the GLRaV-1 coat protein gene using the maximum likelihood estimation (Supplementary Figure S8). The obtained nucleotide sequences were similar to the variants from Bosnia and Herzegovina, Algeria, China and belonged to molecular group III. A BLASTn comparison of the nucleotide sequences of the coat protein gene of the Russian isolates of GLRaV-1 with those available in the GenBank showed that the identity of the Russian isolates was 86.31–94.64%.

Currently, 6 phylogenetic groups of GLRaV-3 are distinguished [23]. In our studies, we found Russian isolates belonging to groups I and II (Supplementary Figure S9). The group I samples were clustered next to isolates from China, South Africa, USA, and Spain, while the group II isolates were clustered next to isolates from China and Greece. The identity of the Russian isolates with GenBank isolates was 99.54–99.77%.

**Figure 4:**
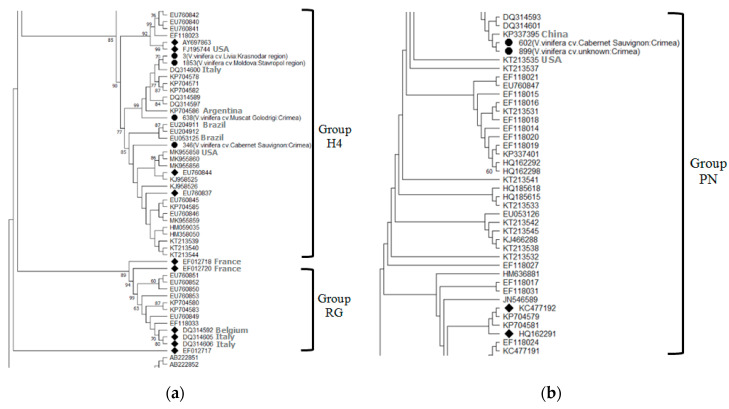
Phylogenetic tree showing the distribution of nucleotide sequences of coat protein genes in Russian isolates of *Grapevine leafroll-associated virus 2* (**a**,**b**) (•) compared to isolates from the GenBank and representative sequences (◆). Geographic origin is indicated for each Russian isolate (in brackets). Bootstrap values > 60% (1000 bootstrap replicates) are shown.

According to previous studies, there are 5 [27] or 6 molecular groups of GLRaV-2 [28]. Phylogenetic analysis of the coat protein gene of Russian isolates revealed the presence of two of them in the southern Russia (Figure 4).

Four GLRaV-2 isolates from commercial grape cultivars Cabernet Sauvignon and Muscat Golodrigi in the Crimea, as well as from table grape cultivars Libya and Moldova in the Krasnodar region and Stavropol region, respectively, belonged to the H4 group. Two isolates found in the vineyards of the Cabernet Sauvignon cultivar and an unknown grape cultivar in the Yalta district of the Republic of Crimea belonged to the PN group. A BLASTn analysis showed the identity of our isolates with isolates from the GenBank at the level of 88.63–99.66%.

An RFLP analysis of two amplicons of the coat protein gene produced different restriction profiles that are presented in Table 1.

Previously, we have determined that the isolate 3 belongs to group 3 of GLRaV-2 (GenBank ID MH074870) by digestion of PCR product amplified with primers V2dCPf2/V2dCPrl [19]. In this study, we confirmed the group of this isolate by digestion of the GLR2CP1/GLR2CP2 fragment. In addition, group 3 also included isolate 638 found on the territory of the Republic of Crimea. Two other Crimean isolates 602 and 899 belonged to group 1a (Supplementary Table S6).

As a result of digestion of two fragments of the GLRaV-2 coat protein gene of isolate 346, we obtained restriction profiles which on the whole did not correspond to any of the previously described groups. For instance, the restriction profile of the GLR2CP1/GLR2CP2 amplicon was the same as the profile of the group 3 isolates. The *Taq*I restriction profile of the V2dCPf2/V2dCPrl amplicon corresponds to the profile of groups 3 and 4, while the *Rsa*I restriction profile—to the profile of the RG group isolates. The data obtained made it possible to suggest the presence of an isolate of a new group in the analyzed sample or the presence of a mixed infection of isolates belonging to several groups of GLRaV-2. At the same time, phylogenetic analysis shows that isolate 346 belongs to the H4 group which is separate from the RG isolates.

PCR with primers V2dCPf2/V2dCPrl did not result in amplification of a fragment of the coat protein gene of isolate 1853 collected from grapevine samples in the Stavropol region. Therefore, we were not able to identify the exact RFLP group by the digestion of two PCR products with the enzymes *Rsa*I and *Taq*I.

## 3. Discussion

Here we describe the results of a large-scale study of the distribution of 7 economically significant grapevine viruses conducted in the southern regions of Russia. For the first time in Russia, we have earlier detected a number of grapevine viruses in individual vineyards of the Crimea and Krasnodar region [16,17,18,19,20]. In this study on the distribution and genetic diversity of viruses, we explored more than three hundred plantations in three regions of Russia; it is noteworthy that in the Stavropol region, no studies on the viral composition of grapevine plantations have previously been conducted. Despite the smaller number of samples collected in Stavropol, which apparently explains the differences in the distribution profile of viruses compared to other regions, 5 of the 7 viruses were detected. This allowed us to include the new isolates in the analysis of genetic diversity of detected viruses. However, the sheltered vineyards of Stavropol region require further phytosanitary monitoring.

The diversity of symptoms of viral diseases that we observed in the process of phytosanitary examination of grapevine plantations is likely to be associated with the difference in action of the same species of viruses on different cultivars and hybrids of grapes. In addition, the size of the territory and the location of vineyards in different zones that we studied indicate the influence of climatic conditions on the symptoms. Infection of the grapevine with several viruses as well as infection with other phytopathogens (fungi, viroids, phytoplasmas, bacteria) can result in unique symptoms and an increase in their manifestation [26,30,31]. Future research should also focus on the distribution in Russian vineyards of other grapevine viruses not included in this monitoring, for example, *Grapevine Pinot gris virus* (GPGV), *Arabis mosaic virus* (ArMV), *Grapevine virus B* (GVB), *Grapevine red blotch-associated virus* (GRBaV), etc.

In this work, we expanded the species composition of viruses for the first time detected in the Krasnodar region; such are GVA, GRSPaV, GLRaV-1, GLRaV-3, GFLV and GFkV. In the Stavropol region, we for the first time detected GVA, GRSPaV, GLRaV-1, GLRaV-2, and GLRaV-3. In the Crimea, we showed the distribution of 7 grapevine viruses: GVA, GRSPaV, GLRaV-1, GLRaV-2, GLRaV-3, GFLV and GFkV.

The data obtained on the prevalence of GRSPaV (in the Crimea, 15% of the collected samples were infected, in the Krasnodar region—10%, in the Stavropol region—6%) indicate its ubiquitous presence in the vineyards of Russia. A phylogenetic analysis showed that most of the studied isolates belong to the BS group and one isolate belongs to the SG1 molecular group. Such clustering did not depend on the geographical location of the plants from which the samples were collected. The SG1 and BS groups and the GRSPaV-1 group have previously been described in Brazil [32]. Isolates from Tunisia have clustered into 4 main groups (I, II, III, IV) and 2 for the first time described groups [33], while phylogenetic studies of Californian isolates have identified the presence of the GRSPaV-SY and GRSPaV-PN groups [34].

The prevalence of GVA varied from 4% in the Krasnodar region to 12% in the vineyards of the Crimea and Stavropol region. Phylogenetic typing confirmed the prevalence of molecular group IV in the vineyards of southern Russia, whereas group I was less prevalent. For comparison, the absolute majority of isolates in Tunisia (70%) belonged to group I, while group IV was the second most prevalent [35]. The prevalence of group I was also reported in Turkey, Iran, and Slovakia [36,37,38]. Analysis of the coat protein sequences of isolates from Washington and California showed that most of them belonged to group III, the second most prevalent group was group I, some of the isolates clustered with isolates of group II and the lowest number of the sequences belonged to group IV [39].

In this work, we studied the distribution of three main pathogens of grapevine leafroll disease: GLRaV-1, GLRaV-2, and GLRaV-3. In general, from 2 to about 10% of the analyzed samples were infected with one or more leafroll viruses at the same time. The data on the distribution of these viruses obtained in this study are in line with the results of our previous studies in the Crimea, when out of 689 samples, 4.9% were found to be infected with GLRaV-1 and 5.4%—with GLRaV-3 [18].

As a result of the study of evolutionary relationships, Russian GLRaV-1 isolates were found to belong to molecular group III. A similar situation was observed in Slovakia, where most of the samples belonged to group III, while several isolates belonged to molecular group I [40]. Isolates found in Turkey clustered into 2 groups [41]. Isolates from China showed a wide genetic diversity and were divided into 5 groups. Most of them belonged to group II, however, the most prevalent in terms of abundance were variants from group I [42].

Our earlier studies of five table cultivars of *Vitis vinifera* allowed to identify GLRaV-2 in about 15% of the samples collected in the Krasnodar region [19]. As a result of the present study, we obtained a more complete picture of the presence of the virus in the vineyards of Russia. GLRaV-2 was detected in 2–6% of the collected samples; the isolates from these samples belonged to the H4 and PN groups. Previously, GLRaV-2 isolates of these groups have been found in vineyards in the USA, along with isolates of the RG group [28].

GLRaV-3 is the predominant species in the vineyards of the Crimea, regardless of the cultivar and age of the grapevine, while GLRaV-1 is predominant in the Stavropol region and Krasnodar region. The overall infection level of GLRaV-3 in the Crimea was about 10%, which is higher than in our previous studies on a smaller selection of samples [18]. The GLRaV-3 isolates that we found belonged to molecular groups I and II which also prevail in the vineyards of Croatia, Portugal, China and New Zealand [43,44,45,46].

Our studies showed that GFkV is widespread almost everywhere; it was identified in 27% of the grape samples collected in the Crimea and in 36% of the samples from the Krasnodar region. The virus has not yet been detected in the Stavropol region. Our results indicating a widespread infection of grapevines with GFkV are supported by data obtained by colleagues from other countries [47]. In Canada, about 30% of the collected samples have been found to be infected with GFkV [48], in Bosnia and Herzegovina—about 25% [49]. Studies in the vineyards of the Balkan Peninsula have found the presence of GFkV in 10% of the samples [50]. In the Czech Republic, 15% of the collected samples have been found to be infected with GFkV [51]. In the vineyards of Slovakia, a high percentage of GFkV infection has also been noted; the identified isolates clustered into two groups and were conditionally divided into 2 molecular groups of the virus [47]. A small amount of information on genetic diversity indicates that this virus has been insufficiently studied as yet, given its potential harmfulness and reduction in berry quality upon its mixed infection with other viruses [14,31]. In our opinion, future research should be aimed at a more detailed study of the genetic diversity of the virus and its pathogenicity.

GFLV is considered one of the most widespread nepoviruses in the world [21]. In our study, this virus was detected in only 2–4% of the samples collected in Krasnodar region and the Republic of Crimea, despite the presence of symptoms in a larger number of samples, which may be associated either with the presence of other viruses, including nepoviruses causing similar symptoms [52] or with the need to use other primers for its detection due to the high recombination of this virus [53]. A phylogenetic analysis of GFLV showed that the detected isolates are not clustered according to geographic origin. On the dendrogram, the Russian isolates were located near isolates from the USA, Tunisia, Italy, Czech Republic, Turkey, and Iran.

The results of phytosanitary monitoring show a wide distribution of viruses in the vineyards of Russia and emphasize the urgent need for a comprehensive approach in the fight against the spread of viral infections. This requires the development and implementation of certification programs for planting material produced in Russia, as well as the control of imported planting material for the presence of key viruses. Creation of nurseries for healthy planting material and implementation of agrotechnical and carrier control measures will make it possible to limit the spread of harmful viruses in Russia. Regular phytosanitary monitoring of main viticulture zones of the south of Russia, including new regions and vineyards, expanding the list of harmful viruses by including recently discovered ones (GPGV, GRBaV etc.) and commonly known (ArMV, GVB etc.) and viroids (*Hop stunt viroid, Grapevine yellow speckle viroid 1 and -2*), improving detection methods are an integral part of the further development of viticulture in Russia.

## 4. Materials and Methods

### 4.1. Sample Collection

In 2014–2019, mostly in August–September, we conducted surveys of vineyards to determine the spread of viruses in the south of Russia. For that purpose, we chose the Krasnodar region and the Republic of Crimea (non-covering cultivation zone), as well as the Stavropol region, where the sum of active temperatures is insufficient for growing grapes without sheltering them for the winter.

At selected vineyards, symptomatic samples presumably infected with viruses were collected. Taking into account that the symptoms of infection and the intensity of their manifestation can vary depending on the cultivar, climatic conditions, pathogen titer in the plant, and coinfection with one or several other viruses, we collected samples with any visual deviations from healthy plants.

Samples of grapevines and leaves were collected from various layers, stored in a refrigerator at +4 °C, and transported to the laboratory for analysis. In the laboratory, samples were stored at −20 °C.

### 4.2. Detection of Grapevine Viruses

Total RNA was extracted from 0.3 g of the combined sample which included fragments of grapevines and leaves, according to a slight modified method described in [54]. RNA quality was verified by electrophoresis in 1% agarose gel stained with ethidium bromide. For cDNA synthesis, we used 2 μL of isolated RNA with Random Hexamer as a primer and RevertAid H Minus Reverse Transcriptase in accordance with the manufacturer’s protocol (Thermo Fisher Scientific, Waltham, MA, USA).

For each sample, a control amplification of 18S rRNA was performed with the primers listed in Supplementary Table S1a.

Viral detection was conducted by PCR using 1 μL of cDNA as a template in a reaction mixture containing *Taq* Buffer with (NH_4_)_2_SO_4_, 0.2 mM each dNTP, 1 μM each primer, 2.5 mM MgCl_2_, 0.375 U *Taq* polymerase (Thermo Fisher Scientific, Waltham, MA, USA). The primers used for detection are listed in Supplementary Table S1a. Some of them were chosen on the basis of literature data, others were designed using the nucleotide sequences of the coat protein genes of viruses available in the NCBI GenBank, the alignment of which was performed using ClustalW in the BioEdit Sequence Alignment Editor v.7.2.0 program.

The PCR amplification consisted of an initial denaturation at 94 °C for 3 min, followed by 35 cycles of 94 °C for 45 s, 56 °C for 45 s, 72 °C for 1 min. The final elongation stage was carried out at 72 °C for 7 min. The resulting PCR products were separated in 1% agarose gel and the corresponding bands were excised. DNA was purified using a Cleanup Standard Kit for DNA purification from agarose gel according to the manufacturer’s protocol (Evrogen, Russia). A virus detected in a single vine was considered as a single isolate. To confirm the presence of virus, the obtained amplicons were sequenced by the Sanger method with forward and reverse primers using Big Dye Terminator v.3.1 chemistry reagents on an ABI PRIZM 3730 automatic sequencer according to the manufacturer’s instructions. The complete sequence was assembled using the BioEdit Sequence Alignment Editor v.7.2.0 software. Then, using the BLASTn algorithm, we compared the sequences that we obtained with those available in the NCBI database. The obtained sequences were deposited in the GenBank, access numbers are listed in Supplementary Table S2.

### 4.3. Phylogenetic Studies

Positive DNA samples were used for sequencing the gene encoding the coat protein and studying the phylogeny of GRSPaV, GVA, GLRaV-1, GLRaV-2, GLRaV-3, GFLV, and GFkV. The list of isolates used for phylogenetic studies is presented in Supplementary Table S3. cDNAs of virus-positive samples were used for PCR with primers for the coat protein gene (Supplementary Table S1b). Amplicons were sequenced in two directions. The resulting nucleotide sequences were used to assemble the coat protein gene. For each virus, we selected in the NCBI database all coat protein sequences which also included the representative sequences of various phylogenetic groups of the viruses under study (Supplementary Tables S4 and S5).

Multiple alignment was performed using ClustalW in the BioEdit Sequence Alignment Editor v.7.2.0 program. Phylogenetic analysis was performed in the MegaX program using the maximum likelihood algorithm [55,56]. The decision whether the obtained isolates belong to one of the molecular groups was made on the basis of their clustering with representative sequences.

For detection of the GLRaV-2 group, we also used RFLP [29]. PCR products amplified with two pairs of primers V2dCPf2/V2CPr1 and GLR2CP1/GLR2CP2 (Supplementary Table S1b) were digested in silico with two restriction enzymes *Rsa*I and *Taq*I using tools of the Clone Manager 6N program. The second pair of primers GLR2CP1/GLR2CP2 was used for more specific detection of the molecular group GLRaV-2RG; comparison of the obtained isolate profiles with those published earlier [29] helped us to decide which one of the 6 GLRaV-2 groups the Russian isolates belonged to.

### 4.4. Recombination Analysis

To search for recombination sites in the genomes of the isolates that we found, we used nucleotide sequences of amplified coat protein genes of viruses GVA, GRSPaV, GLRaV-1, GLRaV-2, GLRaV-3, GFLV, and GFkV, as well as nucleotide sequences of coat protein genes of all viruses submitted to GenBank as of March 2021 (Supplementary Table S5). The search for potential recombination sites and verification of sequence congruity with putative parental forms were conducted using the RDP v.4.100 software [22]. The conclusion about the presence of a recombination point was made based on the programs: RDP, GENECONV, BOOTSCAN, MAXCHI, CHIMAERA, SISCAN, 3SEQ. When analyzing the sequences, the default program settings were used. Only those sites that were identified by four or more programs and had *p* ≤ 0.05 were considered significant.

## Figures and Tables

**Table 1:** RFLP profiles of different GLRaV-2 isolates.

Isolate	Amplicons			Group of Isolates
	GLR2CP1/GLR2CP2	V2dCPf2/V2dCPrl		
	*Taq*I	*Taq*I	*Rsa*I	
3 (MH074870)	I	B	B	3
346	I	B	E	unknown
602	II	A	A	1a
638	I	B	B	3
899	II	A	A	1a
1853	I	-	-	not determined

A, B, E—unique restriction profiles obtained as a result of *Rsa*I or *Taq*I digestion of PCR products amplified with primers V2dCPf2/V2dCPrl. Letter designations correspond to previously published profiles [29]. I, II—unique restriction profiles obtained as a result of *Taq*I digestion of a fragment amplified with primers GLR2CP1/GLR2CP2. “-”—PCR product was not amplified with this pair of primers.

## Data Availability

Representative sequences were deposited in GenBank under the accession numbers: KX348547, MH074870, MW800127–MW800131, MW810491–MW810505, MW916843–MW916865, MW929097, MW929098, MW970077, MZ031985, MZ014522–MZ014525.

## Supplementary Materials

The following are available online at https://www.mdpi.com/article/10.3390/plants10061080/s1, Figure S1: Mixed infection of viruses affected grapevine in the Crimea (percentage of the total number of samples collected in the region). Figure S2: Mixed infection of viruses affected grapevine in the Krasnodar region (percentage of the total number of samples collected in the region). Figure S3: Mixed infection of viruses affected grapevine in the Stavropol region (percentage of the total number of samples collected in the region). Figure S4: Phylogenetic tree showing the distribution of nucleotide sequences of coat protein genes in Russian isolates of *Grapevine virus A.* Figure S5: Phylogenetic tree showing the distribution of nucleotide sequences of coat protein genes in Russian isolates of *Grapevine rupestris stem pitting-associated virus.* Figure S6: Phylogenetic tree showing the distribution of nucleotide sequences of coat protein genes in Russian isolates of *Grapevine fleck virus.* Figure S7: Phylogenetic tree showing the distribution of nucleotide sequences of coat protein genes in Russian isolates of *Grapevine fanleaf virus.* Figure S8: Phylogenetic tree showing the distribution of nucleotide sequences of coat protein genes in Russian isolates of *Grapevine leafroll-associated virus 1.* Figure S9: Phylogenetic tree showing the distribution of nucleotide sequences of coat protein genes in Russian isolates of *Grapevine leafroll-associated virus 3.* Table S1: Primers used for virus detection and for phylogenetic studies. Table S2: List of sequences submitted to the GenBank after validation of detection. Table S3: List of coat protein gene sequences submitted to the GenBank and their identity to the GenBank isolates. Table S4: Representative virus isolates used for group definition. Table S5: Number of coat protein gene sequences used for recombination and phylogenetic analysis. Table S6: RFLP patterns of the GLRaV-2 coat protein gene amplicons [57,58,59,60,61,62,63].
